# Treatment of Bone Metastasis

**DOI:** 10.3390/curroncol29080411

**Published:** 2022-07-22

**Authors:** Costantino Errani

**Affiliations:** III Clinica di Ortopedia e Traumatologia, IRCCS Istituto Ortopedico Rizzoli, 40136 Bologna, Italy; costantino.errani@ior.it

The incidence of metastatic bone disease is increasing, as patients with cancer are living longer [1,2,3]. Bone is the third most common site of metastatic disease, after the lungs and the liver [1,4]. Long bone metastasis is a common presentation in patients with advanced cancer, occurring in up to 70% of patients [1,4]. In 2008, it was estimated that almost 300,000 patients with advanced cancer in the USA had bone metastases [1,4]. Bone metastases can dramatically decrease the quality of life of patients as a result of skeletal-related events [1,5]. In 2006, the financial burden of treating patients with metastatic bone disease in the USA per year was estimated at approximately USD 12.6 billion, accounting for 17% of the total annual cost of cancer treatments [5]. Patients with metastatic bone disease may seek medical care at community hospitals [1,3]. Traditional management techniques involve a combination of pharmacotherapy, radiotherapy and surgical procedures [1]. Over the last few decades, advances in medical and surgical treatments have been proposed regarding the management of metastatic bone disease [1]. Considering the limited expectancy of most patients with bone metastasis, the main goal of novel medical and minimally invasive treatments is to improve the quality of life of patients with bone metastases and to reduce the adverse effects related to traditional medical and surgical treatments [1]. This Special Issue on the “Treatment of Bone Metastasis” will be useful to guide orthopedic surgeons in their decision-making regarding treatment approaches for patients with bone metastasis. The life expectancy of patients with bone metastasis seems to be the most important factor in determining surgical treatment and avoiding over or under treatment [6]. Consequently, several studies have been conducted on prognostic factors affecting survival [6,7,8,9,10]. Ben-Gal et al. evaluated each model’s performance, assessing the estimated discriminative power and calibration accuracy for patients with bone metastases. Among externally validated survival prediction scores, the PathFx model, SPRING and Optimodel were found to be the best models in terms of performance [11]. These data contribute to increasing our knowledge on the prognostic scores of patients with bone metastasis that have already been published previously [12,13]. Meares et al. found that the Optimodel demonstrated the highest accuracy for predicting 12-month (area under the curve (AUC) = 0.79) and 24-month (AUC = 0.77) survival. The PathFx model was the most accurate at predicting 3-month survival (AUC = 0.70) and 6-month (AUC = 0.70) survival [12]. Alfaro et al. compared the performance of different survival prognostic models on patients with long bone metastases in a Chilean population [13]. The PathFx score model demonstrated the highest accuracy when predicting a survival time of 3 or 6 months. The IOR score model was the most accurate measure at predicting a survival time of 12 months [13]. Regarding the treatment of bone metastasis, Mollica et al. remind us that systemic treatment remains the main treatment for delaying skeletal-related events and that the use of bone-targeting agents consisting of bisphosphonates and denosumab is an essential part of the treatment of metastatic bone disease [14]. Metastatic bone disease can cause debilitating pain, pathologic fractures, and reduced quality of life [15]. The goals of surgical treatment are to provide pain relief and return the function of the bones with a construct that provides stability to allow for immediate weight bearing [15]. Current surgical treatment options include intramedullary nail fixation, hemiarthroplasty or total hip arthroplasty, and megaprosthetic reconstructions [15]. Most intramedullary nail fixation-related complications usually occur more than one year after treatment, in contrast to prosthetic-related complications that occur earlier [1,6]. Mahdal et al. evaluated the implant survival, functional score and complications of intercalary megaprostheses implanted for metastatic bone disease of the femoral and humeral diaphysis [16]. There was a significantly higher risk of the aseptic loosening of the intercalary megaprosthesis in the humerus compared with that in the femur (odds ratio 13.79, 95% confidence interval 1.22–151.05, *p* = 0.0297). The overall cumulative implant survival was 92% at 1 year after surgery and 72% at 5 years after surgery [16]. Thorkildsen et al. performed a comparative analysis of complications and revision surgery for patients with metastatic bone disease and patients with bone sarcoma treated with megaprosthesis [17]. The rate of revision surgery was significantly lower for patients with bone metastasis (8% at 1 year, 12% at 2 years) compared to patients with bone sarcoma (18% at 1 year, 24% at 2 years) (*p* = 0.04) [17]. The results of these studies suggest that, in consideration of the high risk of complications related to megaprosthesis and the consequent need for revision surgery, patients with a life expectancy less than 12 months should be treated with less invasive surgery, such as intramedullary nail fixation, or minimally invasive treatments such as embolization, thermal ablation therapy, high-intensity focused ultrasound or electrochemotherapy [1,6,18,19,20]. Pusceddu et al. analyzed 35 patients with 41 vertebral spinal metastases who underwent radiofrequency ablation associated with vertebral augmentation [21]. The mean visual analog scale score dropped from 5.7 (95% CI 4.9–6.5) to 0.9 (95% CI 0.4–1.3) (*p* < 0.001). The decrease in visual analog scale score, following radiofrequency ablation, remained constant over time for up to one year, suggesting that pain relief was immediate and durable [21]. Faiella et al. evaluated the impact of an augmented reality navigation system for percutaneous biopsies and ablative treatments on bone lesions, showing that the use of the augmented reality navigation system reduced the number of computer tomography scans, procedural time and patient’s radiation dose [22]. Campanacci et al. treated 38 patients with bone metastases using electrochemotherapy [23]. The tumor response were assessed as per Evaluation Criteria in Solid Tumors: 25% of patients had an objective response, 59% of patients had a stable disease and 16% of patients had progressive disease [23]. In patients with bone metastasis, especially if the patient has a limited expected survival, the indications for surgical treatment are limited [24]. Immediate pain relief and an improvement in functional status are important, and treatment complications are unwanted [24]. Minimally invasive techniques seem to be effective for both pain relief and local tumor control, suggesting that their use may increase in the near future [18,20,24]. The goal of the management of patients with metastatic bone disease is pain relief and an improvement in the quality of remaining life [25]. This Special Issue on the “Treatment of Bone Metastasis” reviews recent findings on the prognosis and treatment of patients with metastatic bone disease in the hopes of stimulating the scientific community to continue research on novel, less invasive therapies.

## References

[1] Errani C., Mavrogenis A.F., Cevolani L., Spinelli S., Piccioli A., Maccauro G., Baldini N., Donati D. (2016). Treatment for long bone metastases based on a systematic literature review. Eur. J. Orthop. Surg. Traumatol. Orthop. Traumatol..

[2] Cheung F.H. (2014). The practicing orthopedic surgeon’s guide to managing long bone metastases. Orthop. Clin. N. Am..

[3] Weber K.L., Randall R.L., Grossman S., Parvizi J. (2006). Management of lower-extremity bone metastasis. J. Bone Jt. Surg. Am..

[4] Coleman R.E. (2006). Clinical features of metastatic bone disease and risk of skeletal morbidity. Clin. Cancer Res. Off. J. Am. Assoc. Cancer Res..

[5] Schulman K.L., Kohles J. (2007). Economic burden of metastatic bone disease in the U.S. Cancer.

[6] Errani C., Cosentino M., Ciani G., Ferra L., Alfaro P.A., Bordini B., Donati D.M. (2021). C-reactive protein and tumour diagnosis predict survival in patients treated surgically for long bone metastases. Int. Orthop..

[7] Forsberg J.A., Eberhardt J., Boland P.J., Wedin R., Healey J.H. (2011). Estimating survival in patients with operable skeletal metastases: An application of a bayesian belief network. PLoS ONE.

[8] Katagiri H., Okada R., Takagi T., Takahashi M., Murata H., Harada H., Nishimura T., Asakura H., Ogawa H. (2014). New prognostic factors and scoring system for patients with skeletal metastasis. Cancer Med..

[9] Willeumier J.J., van der Linden Y.M., van der Wal C.W.P.G., Jutte P.C., van der Velden J.M., Smolle M.A., van der Zwaal P., Koper P., Bakri L., de Pree I. (2018). An Easy-to-Use Prognostic Model for Survival Estimation for Patients with Symptomatic Long Bone Metastases. J. Bone Jt. Surg. Am..

[10] Ratasvuori M., Wedin R., Hansen B.H., Keller J., Trovik C., Zaikova O., Bergh P., Kalen A., Laitinen M. (2014). Prognostic role of en-bloc resection and late onset of bone metastasis in patients with bone-seeking carcinomas of the kidney, breast, lung, and prostate: SSG study on 672 operated skeletal metastases. J. Surg. Oncol..

[11] Ben-Gal O., Soh T.C.F., Vaughan S., Jayasanker V., Mahendra A., Gupta S. (2022). The Prediction of Survival after Surgical Management of Bone Metastases of the Extremities—A Comparison of Prognostic Models. Curr. Oncol..

[12] Meares C., Badran A., Dewar D. (2019). Prediction of survival after surgical management of femoral metastatic bone disease-A comparison of prognostic models. J. Bone Oncol..

[13] Alfaro P.A., Delgado J., Dumas A., Mesa C., Wevar O., Herrera C., Padilla F., Botello E. (2021). Comparison between different prognostic models to be used for metastatic bone disease on appendicular skeleton in a Chilean population. Eur. J. Orthop. Surg. Traumatol. Orthop. Traumatol..

[14] Mollica V., Nuvola G., Tassinari E., Nigro M.C., Marchetti A., Rosellini M., Rizzo A., Errani C., vMassari F. (2022). Bone Targeting Agents in Patients with Prostate Cancer: General Toxicities and Osteonecrosis of the Jaw. Curr. Oncol..

[15] Axelrod D., Gazendam A.M., Ghert M. (2021). The Surgical Management of Proximal Femoral Metastases: A Narrative Review. Curr. Oncol..

[16] Mahdal M., Pazourek L., Apostolopoulos V., Adámková Krákorová D., Staniczková Zambo I., Tomáš T. (2022). Outcomes of Intercalary Endoprostheses as a Treatment for Metastases in the Femoral and Humeral Diaphysis. Curr. Oncol..

[17] Thorkildsen J., Strøm T.A., Strøm N.J., Sellevold S., Norum O.-J. (2022). Megaprosthesis for Metastatic Bone Disease-A Comparative Analysis. Curr. Oncol..

[18] Tsukamoto S., Kido A., Tanaka Y., Facchini G., Peta G., Rossi G., Mavrogenis A.F. (2021). Current Overview of Treatment for Metastatic Bone Disease. Curr. Oncol..

[19] Gazendam A., Axelrod D., Wilson D., Ghert M. (2021). Emerging Concepts in the Surgical Management of Peri-Acetabular Metastatic Bone Disease. Curr. Oncol..

[20] Papalexis N., Parmeggiani A., Peta G., Spinnato P., Miceli M., Facchini G. (2022). Minimally Invasive Interventional Procedures for Metastatic Bone Disease: A Comprehensive Review. Curr. Oncol..

[21] Pusceddu C., De Francesco D., Melis L., Ballicu N., Fancellu A. (2021). The Role of a Navigational Radiofrequency Ablation Device and Concurrent Vertebral Augmentation for Treatment of Difficult-to-Reach Spinal Metastases. Curr. Oncol..

[22] Faiella E., Castiello G., Bernetti C., Pacella G., Altomare C., Andresciani F., Beomonte Zobel B., Grasso R.F. (2021). Impact of an Augmented Reality Navigation System (SIRIO) on Bone Percutaneous Procedures: A Comparative Analysis with Standard CT-Guided Technique. Curr. Oncol..

[23] Campanacci L., Cevolani L., De Terlizzi F., Saenz L., Alì N., Bianchi G., Donati D.M. (2022). Electrochemotherapy Is Effective in the Treatment of Bone Metastases. Curr. Oncol..

[24] Errani C., Bazzocchi A., Spinnato P., Facchini G., Campanacci L., Rossi G., Mavrogenis A.F. (2019). What’s new in management of bone metastases?. Eur. J. Orthop. Surg. Traumatol. Orthop. Traumatol..

[25] Tsukamoto S., Errani C., Kido A., Mavrogenis A.F. (2021). What’s new in the management of metastatic bone disease. Eur. J. Orthop. Surg. Traumatol. Orthop. Traumatol..

